# Syntaxin 3B: A SNARE Protein Required for Vision

**DOI:** 10.3390/ijms251910665

**Published:** 2024-10-03

**Authors:** Himani Dey, Mariajose Perez-Hurtado, Ruth Heidelberger

**Affiliations:** Department of Neurobiology and Anatomy, McGovern Medical School at the University of Texas Health Science Center at Houston, Houston, TX 77030, USA; himani.dey@uth.tmc.edu (H.D.);

**Keywords:** syntaxin, stx3, retina, ribbon synapse, SNARE, retinal degeneration, exocytosis, membrane fusion, retinal dystrophy, syntaxin 3B

## Abstract

Syntaxin 3 is a member of a large protein family of syntaxin proteins that mediate fusion between vesicles and their target membranes. Mutations in the ubiquitously expressed syntaxin 3A splice form give rise to a serious gastrointestinal disorder in humans called microvillus inclusion disorder, while mutations that additionally involve syntaxin 3B, a splice form that is expressed primarily in retinal photoreceptors and bipolar cells, additionally give rise to an early onset severe retinal dystrophy. In this review, we discuss recent studies elucidating the roles of syntaxin 3B and the regulation of syntaxin 3B functionality in membrane fusion and neurotransmitter release in the vertebrate retina.

## 1. Syntaxin 3B Overview

Syntaxin 3B belongs to a family of proteins that play key roles in intracellular membrane trafficking [1]. The canonical family member syntaxin 1 has an essential role in the exocytotic release of neurotransmitter at conventional synapses [2,3]. At these synapses, syntaxin 1, located on the presynaptic plasma membrane, interacts via its soluble N-ethylmaleimide-sensitive factor attachment protein receptor (SNARE)-binding domain with the SNARE domains of SNAP-25, a protein associated with the presynaptic plasma membrane, and the SNARE domain of synaptobrevin/VAMP (vesicle-associated membrane protein), a protein located on the synaptic vesicle membrane, to form a tripartite protein complex known as the SNARE complex that bridges the two membranes. This SNARE complex constitutes the minimal protein machinery required for membrane fusion [4,5]. In addition to its well-established role in exocytosis, syntaxin 1 may also be required for membrane fusion events associated with neuronal viability, axonal outgrowth, and the organization of synaptic ultrastructure [6,7,8,9].

**Figure 1:**
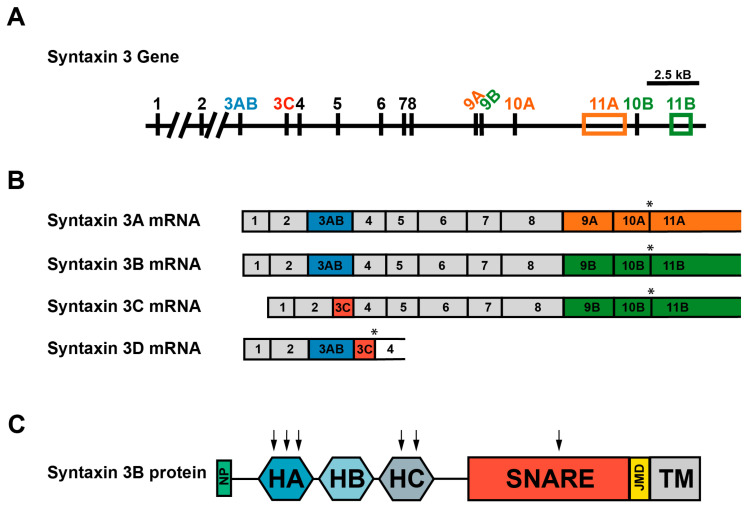
The syntaxin 3 gene and its splice forms. (**A**) Syntaxin 3 gene structure. The exons utilized by the various splice forms are as indicated. (**B**) mRNA transcripts corresponding to the different syntaxin 3 splice forms. Exons are color-coded to indicate common regions shared by the different splice forms. * indicates the position for the stop codon in the transcript. (**C**) Domain organization of the syntaxin 3B protein. The N-terminal peptide (NP) is located at the N-terminus of the protein, followed by the three helices HA, HB, HC (comprising the Habc domain), the soluble N-ethylmaleimide-sensitive factor attachment protein receptor (SNARE)-binding domain, the juxtamembrane domain (JMD), and the transmembrane (TM) domain at the C-terminus. Each arrow indicates the approximate position of a point mutation identified in human patients with an early onset, severe retinal dystrophy (EOSRD). (Adapted from Janecke et al., 2021, Figure 1 [13].)

The retina-specific syntaxin 3 splice form, syntaxin 3B, is encoded by the syntaxin 3 gene through differential splicing [1,10,11]. The two major splice forms of syntaxin 3, syntaxin 3A and 3B, are identical in the N-terminal half of the protein due to their sharing of exons 1–8, but they differ in the C-terminal half of the SNARE binding domain and in the transmembrane domain due to differences in exons 9–10 (Figure 1A,B) [10,12,13]. The differences in the latter half of the protein raise the possibility that syntaxins 3A and 3B interact with different SNARE binding partners and may target to different membranes. In this context, it is interesting to note that syntaxin 3B shares a greater (67%) sequence homology with syntaxin 1A than does syntaxin 3A [10,12], and both syntaxin 1A and syntaxin 3B form functional SNARE complexes with SNAP-25 and VAMP2 [5,10,14]. By contrast, syntaxin 3A is expressed in polarized epithelial cells and non-neuronal secretory cells throughout the body, including in the gastrointestinal, urinary, and immune systems, where syntaxin 3A is typically involved in basal to apical trafficking and regulated secretion [15,16,17,18]. In addition, syntaxin 3 has been postulated to be present in the postsynaptic spines of conventional synapses and to have an important role in some types of synaptic plasticity [19,20,21,22]. Conservation of the N-terminus between syntaxins 3A and 3B suggests that phosphorylation at T14, which has been shown to regulate the ability of syntaxin 3B to form SNARE complexes [23,24] and will be discussed in more detail in this review, also regulates the formation of syntaxin 3A-containing complexes. Given the wide distribution of syntaxin 3A in the body, it is not surprising that mutations in the syntaxin 3 gene can have devastating consequences for human health [13,25,26,27]. Furthermore, when these mutations additionally disrupt syntaxin 3B function, human vision is compromised [13].

There are also two minor isoforms of syntaxin 3 whose roles and localization are not well elucidated. Syntaxin 3C differs from syntaxin 3B in one exon, while syntaxin 3D encodes for a truncated protein that lacks both the SNARE and transmembrane domains required for membrane fusion (Figure 1A,B) [11]. Interestingly, there is evidence to suggest that the latter may act as a transcriptional regulator [28]. The minor isoforms will not be discussed further in this review.

We note that many of the earlier published studies simply refer to “syntaxin 3” rather than a particular splice form. Indeed, syntaxin 3B was not widely recognized until it was identified as a retina-specific splice form by EST-database searches combined with tissue specific RT-PCR approaches, characterized using the GST pull-down approach in the mouse retina [10], and confirmed in retinal bipolar cells using a combination of single-cell RT-PCR, quantitative real time RT-PCR, and in situ hybridization [10,12]. In addition, due to the high sequence homology in exons 1–8 [10], there is a paucity of syntaxin 3 isoform-specific tools that distinguish between syntaxin 3A and 3B. For example, one commercially available antibody marketed as a highly specific syntaxin 3B antibody has been shown to recognize both syntaxin 3A and syntaxin 3B [29]. In addition, the syntaxin 3B-derived peptide used to inhibit the formation of new syntaxin 3B-containing SNARE complexes in the retina [12,30,31] is expected to also inhibit the formation of new syntaxin 3A-containing SNARE complexes in cells that express syntaxin 3A. In this review, we use the terms “syntaxin 3A” or “syntaxin 3B” in situations in which the syntaxin 3 splice form is known, such as in biochemical experiments, or the major isoform in a particular cell has been identified.

## 2. Syntaxin 3B and Retinal Ribbon-Style Synapses

Retinal photoreceptors and bipolar cells are required for natural, image-forming vision. Photoreceptors transduce the absorption of a photon of light into an electrical signal that is conveyed via chemical synaptic transmission to downstream neurons by the calcium-dependent release of glutamate. Retinal bipolar cells receive this information from photoreceptors and provide the main throughput pathway for the flow of visual information to third-order retinal neurons. They also release the neurotransmitter glutamate. Both classes of neurons are specialized for continuous neurotransmitter release that is graded with stimulus intensity [32,33,34,35,36,37]. To support this unusual mode of release, their active zones have a unique architecture that is marked by the presence of the synaptic ribbon, an electron-dense, sheet-like structure to which a large number of closely packed synaptic vesicles tether in an orderly array [38,39,40]. In the simplest scenario, vesicles located on the lowermost rows of the ribbons are in contact with the plasma membrane, where they form the SNARE complexes required for fast, stimulus-evoked neurotransmitter release, while those vesicles further up the ribbon resupply this fast-releasing pool of fusion-competent vesicles [31,37,38]. In addition to their distinct synaptic architecture, retinal ribbon-style synapses differ from conventional synapses at the molecular level, expressing unusual isoforms of several presynaptic proteins that support their unusual signaling abilities [37,38,41,42].

**Figure 2:**
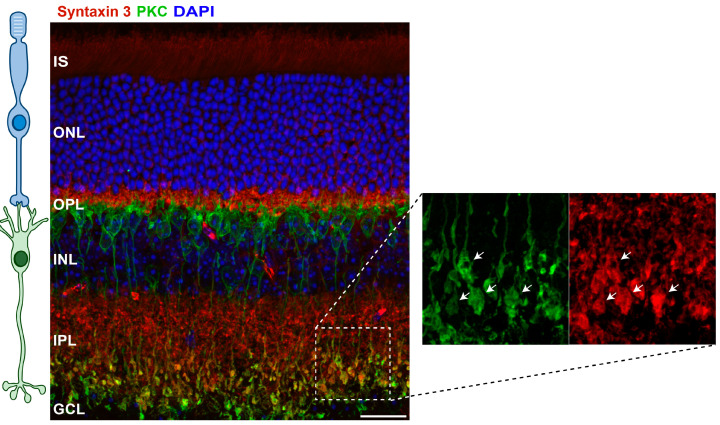
Syntaxin 3B distribution in the mouse retina. Vertical section of mouse retina immunolabeled with an antibody raised against syntaxin 3B (red) ([49]; but see Campbell et al. [29] for additional characterization), PKC, a marker for rod bipolar cells (green), and the nuclear stain DAPI (blue). Syntaxin 3 immunolabeling is enriched in the two synaptic layers, the outer plexiform layer (OPL), where the synaptic terminals of the rod and cone photoreceptors reside, and the inner plexiform layer (IPL), where bipolar cell synaptic terminals reside. The cartoon on the left depicts the approximate positioning of a rod photoreceptor and a postsynaptic rod bipolar cell. Scale bar = 20 um. Abbreviations: IS (inner segment), ONL (outer nuclear layer), OPL (outer plexiform layer), INL (inner nuclear layer) IPL (inner plexiform layer), and GCL (ganglion cell layer). OS (outer segment) is not marked. Inset: An expanded view of the distal IPL. Arrows point to some of the many PKC-positive rod bipolar cell synaptic terminals. Note that each of the rod bipolar terminals immunolabels for syntaxin 3.

One of these unusual presynaptic protein isoforms is syntaxin 3B. Morgans et al., 1996 [43], first identified syntaxin 3 as a SNARE protein in the retina through the isolation of SNARE complexes in retinal extract that contained syntaxin 3 complexed with SNAP-25 (synaptosomal-associated protein, 25 kDa) and VAMP2 (vesicle-associated membrane protein 2). Using an immunohistochemical approach, they further showed that syntaxin 3 was highly expressed in the synaptic boutons of photoreceptors and bipolar cells [43], a result that has been convincingly replicated in multiple vertebrate species, including in mouse (e.g., Figure 2) and human retinae [10,12,13,44,45]. By contrast, other plasma membrane syntaxins, including the conventional presynaptic syntaxin, syntaxin 1, were expressed at very low levels in these neurons, if at all [42,45,46,47,48]. Further investigations using single-cell PCR approaches, retinal transcript expression analysis, and in situ hybridization subsequently established the presence of syntaxin 3B in photoreceptors and bipolar cells of the fish, mouse, and human retinae [10,12,13]. That syntaxin 3B catalyzes membrane fusion in partnership with SNAP-25 and VAMP2 has been confirmed with the use of well-controlled, in vitro proteoliposomal fusion assays [10,14].

The identification of syntaxin 3B as a retinal t-SNARE protein [10,12,43], combined with the prominent localization of syntaxin 3 immunolabeling in the synaptic layers of the vertebrate retina, the outer plexiform layer (OPL), and the inner plexiform layer (IPL) (e.g., Figure 2) and the abundance of retinal syntaxin 3B mRNA expression versus the paucity of retinal syntaxin 3A mRNA [10,12,13] has led to the assertion that syntaxin 3B plays a critical role in stimulus-evoked glutamate release at retinal ribbon-style synapses. This hypothesis was put to the test in experiments that monitored neurotransmitter release from living photoreceptors and bipolar cells using electrophysiological approaches. To disrupt syntaxin 3B function, these experiments capitalized on the use of a short peptide, derived from the N-terminal region of the syntaxin 3B SNARE domain and shown to compete with full-length syntaxin 3B, to inhibit the formation of new syntaxin 3B-containing SNARE complexes in the retina [12,31]. When acutely introduced into retinal photoreceptors or bipolar cells via the patch-clamp recording electrode, an initial secretory response was preserved [12,31], indicating that some synaptic vesicles had already formed the necessary SNARE complexes for membrane fusion prior to introduction of the inhibitory peptide, while a secondary, later component of release that required the formation of new, syntaxin 3B-containing SNARE complexes, was inhibited [31]. What was surprising was that the initial response was not limited to the smaller number of vesicles located on the lower rows of the synaptic ribbons that directly contact the plasma membrane. Rather, the magnitude of the response suggested that the totality of the ribbon-associated pool of synaptic vesicles, including those not in direct contact with the plasma membrane, had formed the SNARE complexes necessary for fusion prior to introduction of the peptide [31]. These results were observed in both photoreceptors and bipolar cells and with the use of two different electrophysiological assays of exocytosis [31]. In the simplest interpretation, these studies established a role for syntaxin 3B in neurotransmitter release at retinal ribbon-style synapses. In addition, these results extended earlier studies of retinal ribbon-style synapses, the results of which indicated that more than just the bottom-most row of vesicles on the synaptic ribbons can become molecularly prepared for rapid, stimulus-evoked release, at least under some conditions [38,50,51].

**Figure 3:**
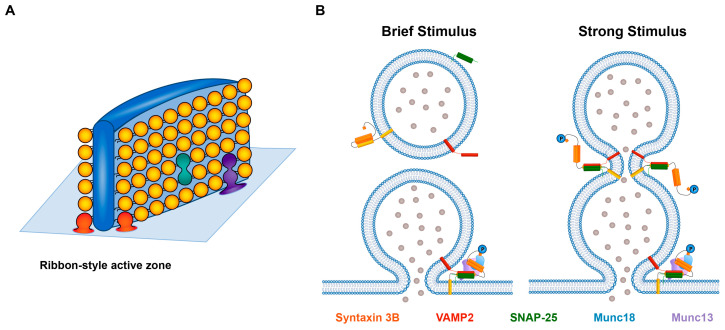
Proposed modes of release at a retinal ribbon-style synapse. (**A**) Schematized version of a synaptic ribbon at the presynaptic plasma membrane with its associated halo of synaptic ribbons (adapted from Matthews 1996, Figure 2C [70]). An individual vesicle at the base of the ribbon forms SNARE complexes that bridge the vesicle and plasma membranes, allowing the vesicle to undergo fusion with the plasma membrane in response to a brief stimulus (one red vesicle). If the fusions of two vesicles are coordinated in time (two red vesicles), this would give rise to MVR. Two vesicles could potentially undergo intervesicular fusion (depicted by the green vesicles) prior to fusing with the plasma membrane to give rise to MVR when the resultant, large vesicle fuses with the plasma membrane (not depicted). Sequential compound fusion may occur when a first vesicle fuses with the plasma membrane and another vesicle fuses to the first vesicle to release its contents into the extracellular space (shown in purple). (**B**) Left side: in response to a brief stimulus, SNARE complexes form between a vesicle and the plasma membrane, facilitated by the phosphorylation of syntaxin 3B at T14. Right side: in response to a stronger stimulus with its larger influx of calcium, syntaxin 3B is phosphorylated on a vesicle at the plasma membrane and on a second vesicle that does not directly contact the plasma membrane. Intervesicular SNARE complexes formed between this second vesicle and the previously fused vesicle allow the second vesicle’s contents to empty into the extracellular space. SNAP-25 and syntaxin 3B, in addition to VAMP2, are shown as being present on synaptic vesicles, consistent with experimental results [23,71].

Syntaxin 3B, in addition to catalyzing fusion between a single vesicle and the plasma membrane (Figure 3A, red vesicles, and Figure 3B, left panel), has also been suggested to catalyze a specialized mode of release called multivesicular release (MVR) [30]. MVR is said to occur when a fusion event has characteristics representative of the equivalent of two or more vesicles simultaneously fusing with the plasma membrane [52]. In the retina, MVR has been proposed to reduce noise and improve the ability to encode event amplitude and contrast coding [30,53,54]. However, there is a debate as to the precise nature of MVR. MVR events could represent the coordinated release of two or more individual synaptic vesicles (Figure 3A, red vesicles) [55]. It could also arise from the fusion of two or more vesicles with each other via an intervesicular fusion reaction (“compound fusion”) that occurs prior to fusion with the plasma membrane (Figure 3A, green vesicles). That some form of compound fusion occurs at retinal ribbon-style synapses has been suggested multiple times over the past two decades [38,56,57]. Perhaps the most compelling argument to support the intervesicular fusion hypothesis at a retinal ribbon-style synapse, and, in particular, sequential compound fusion, wherein a vesicle fuses with a vesicle that has already fused with the plasma membrane, comes from a very elegant, live-cell, super-resolution imaging study of vesicle movement and fusion along synaptic ribbons in fish retinal bipolar cells. This study demonstrated that vesicles can fuse and become exposed to the extracellular environment mid-ribbon and far from the expected position of the plasma membrane [58], a finding consistent with sequential compound fusion (Figure 3A, purple vesicles).

MVR at photoreceptor terminals was shown recently to be particularly dependent upon the ability to form new syntaxin 3B-containing SNARE complexes [30]. That syntaxin 3B could act as a retinal SNARE protein for intervesicular fusion events at a retinal ribbon-style synapse is a novel concept that offers a first glimpse into the possible molecular machinery of intervesicular MVR at retinal ribbon-style synapses [30,31,52]. This hypothesis is bolstered by studies of exocytosis in non-neuronal secretory cells, where it is well established that syntaxin 3 is present on secretory granules and other intercellular membranes and plays a key role in intra-vesicular fusion events [15,16,59,60,61,62,63,64,65]. In mast cells, for example, syntaxin 3 is located on both secretory granules and the plasma membrane [66] and is required for the fusion of secretory granules with the plasma membrane, the fusion of granules with each other prior to their fusion with the plasma membrane, and the fusion of individual granules with granules that have already fused with the plasma membrane [60]. The latter mode of release, called sequential compound fusion, allows for quantized release to continue without the need to first clear the plasma membrane. In photoreceptors and bipolar cells, sequential compound fusion (Figure 3B) could allow for continuous, quantal neurotransmitter release under conditions in which membrane retrieval and clearance were inhibited, such as during periods of strong stimulation and elevated intraterminal calcium [34,35,36,67,68,69].

SNARE-mediated inter-vesicular MVR and sequential quantal release at retinal ribbon-style synapses would minimally require that syntaxin 3B be present on synaptic vesicles. Subcellular fractionation experiments performed in mouse retina have revealed that while there is a strong syntaxin 3B signal in the plasma membrane fraction, the synaptic vesicle fraction also contains significant levels of syntaxin 3B and SNAP-25, in addition to the expected VAMP2 [23]. Even at conventional synapses, there is a small amount of syntaxin 1 present on synaptic vesicles [71]. Given that only a few SNARE complexes are required to drive synaptic vesicle fusion [72,73,74] and that vesicles on synaptic ribbons are densely clustered [32,38,39], it seems plausible that syntaxin 3B-containing SNARE complexes could form between vesicles tethered to the synaptic ribbons [30,31].

## 3. Non-Synaptic Roles of Syntaxin 3B

Syntaxin 3B is also expressed in photoreceptor and bipolar cell somata and in photoreceptor outer segments, where it is important for membrane fusion events associated with trafficking through the Golgi network and for photoreceptor renewal. With respect to the latter, syntaxin 3B has been implicated in the delivery of inner segment proteins to the outer segments of both rod and cone photoreceptors and to support disk and outer segment morphogenesis in the rod photoreceptor [75,76]. For example, in the rod photoreceptor, syntaxin 3B is expressed in the inner segment plasma membrane near the connecting cilia, where it has been proposed to act as a SNARE protein that catalyzes the fusion of vesicles containing rod outer segment membrane proteins with disk membranes [49,75,77]. The neuronal SNARE binding partner SNAP-25 is also found at this location, as is the non-canonical VAMP, VAMP7 [77,78]. SNAP-25 has recently been reported to be essential for photopigment trafficking and survival in both rods and cones [79]. Syntaxin 3B has also been suggested to act in concert with a retinal-specific form of REEP6 (receptor expressing enhancing protein 6), a protein required for rod survival, where it may traffic a particular subset of vesicles to the rod plasma membrane [80]. Those interested in a more thorough discussion of the non-synaptic roles of syntaxin 3 in photoreceptors are referred to a recent review [81].

## 4. Inactivation of the Syntaxin 3 Gene in Mice

In mice, global inactivation of the syntaxin 3 gene is embryonic lethal [13,60]. Inactivation of the syntaxin 3 gene in a cell-specific manner selectively in mouse rod photoreceptors or the almost complete knockdown of syntaxin 3 in mouse rods leads to the mislocalization of rod outer segment proteins such as rhodopsin, the rod photopigment, and protein peripherin 2, a protein that may serve as a structural protein in the outer segment [13,44,49,75]. In the absence of sufficient syntaxin 3, not only do these particular outer segments proteins not reach their target destinations, they become mislocalized to other cellular compartments, including the synaptic terminal [13,44]. Inactivation of the syntaxin 3 gene selectively in rod photoreceptors leads to their rapid degeneration, followed by the secondary loss of cone photoreceptors, and the eventual loss of virtually the entire outer nuclear layer of the retina [13,44]. However, a single copy of the syntaxin 3 gene in mouse rod photoreceptors is sufficient to maintain viability and preserve the outer nuclear layer [44,82].

Inactivation of the syntaxin 3 gene in photoreceptors would also be expected to negatively impact neurotransmitter exocytosis. Indeed, loss of syntaxin 3 expression in mouse photoreceptors was associated with multiple changes in active zone ultrastructure, including the presence of misshapen and detached ribbons, with rods being affected to a greater extent than cones [44]. In addition, the amplitude of the electroretinogram (ERG) b wave, representing electrical activity in the bipolar cells, was decreased following inactivation of the syntaxin 3 gene in photoreceptors prior to a significant decrease in photoreceptor number [44]. Either a decrease in phototransduction or synaptic transmission could account for the observed b-wave decrement. Thus, the extent to which altered presynaptic function contributes to the observed defects under these conditions remains to be determined. However, the possibility of a defect in synaptic transmission at the ribbon-style synapses of photoreceptors and bipolar cells must be considered when presented with a visual impairment of unknown cause that originates from within the retina.

## 5. Retinal Dystrophy and Mutations in the Human Syntaxin 3 Gene

Pathogenic mutations in the syntaxin 3 gene have recently been identified in human subjects. Biallelic loss-of-function mutations in the human syntaxin 3 gene affecting syntaxin 3A were identified that gave rise selectively to microvillus inclusion disorder (MVID), a severe, inherited gastrointestinal disease that presents in early infancy and has a poor prognosis [13,25,26,27]. Vision in these young patients was reported to be normal. However, when an exon shared between syntaxin 3A and 3B contained the pathogenic mutation, these young MVID patients additionally exhibited an early-onset, severe retinal dystrophy (EOSRD) [13], highlighting the importance of syntaxin 3B for human vision. Visual impairments were evident as early as five to six months of age, and they were progressive. Affected children struggled to locate, track, and reach for objects, half were reported to have nystagmus (involuntary oscillations in eye movement), and when performed, fundoscopic examination revealed pallor of the optic discs [13]. Electrophysiological testing, performed on several of the children, demonstrated a severe rod and cone photoreceptor dysfunction, with older children exhibiting little to no detectable responses to light [13]. The presence of small ERG responses early in the course of the disease raises the possibility that a different plasma membrane syntaxin helps support the initial formation, survival, and function of photoreceptors that is subsequently replaced by syntaxin 3B. The importance of syntaxin 3B over syntaxin 3A for human visual function is further supported by an analysis revealing that more than 90% of the total syntaxin 3 mRNA in a human retina expression data set [83] corresponds to syntaxin 3B [13], while syntaxin 3A mRNA makes up only about 1%, in good agreement with earlier findings in the mouse retina [10].

The mutations associated with this EOSRD produced a premature stop codon that mapped either to the Habc domain of syntaxin 3 or to the SNARE domain (Figure 1C) [13]. The gene products are thought to be eliminated from the cells either through mRNA guided decay or protein degradation pathways, resulting in a loss-of-function phenotype [13]. In this context, it is interesting to note that there is a poly-ubiquitination site in the Habc domain at K63 [84]. Conventionally, polyubiquitination at lysine residues is linked to proteasomal degradation of proteins. It is conceivable that the K63 residue of truncated syntaxin 3B proteins might act as a signal for proteasome-mediated degradation. This supposition also implies that modifications targeting other domains in the full-length syntaxin 3B might exist that prevent degradation. However, at present, the significance of this K63 site in the context of visual function remains to be determined. Even if not degraded, the produced gene products associated with the human syntaxin 3 mutations would not be expected to catalyze membrane fusion because they lack most or all of the SNARE domain and the transmembrane domain. For similar reasons, monoallelic loss-of-function mutations at these sites would be unlikely to have a dominant negative impact on SNARE complex formation. Consistent with this assertion, the parents of these children, who each have a monoallelic pathogenic syntaxin 3 mutation, were not reported to have gastrointestinal symptoms or visual deficits at the time that their children presented to the clinic [13].

How many alleles of the syntaxin 3 gene are required to maintain normal functionality? As noted above, one normal allele appears to be sufficient to support vision and human health, at least through the reproductive years. The extent to which a single copy of the syntaxin 3 gene is sufficient to maintain photoreceptor viability in the aging retina was addressed experimentally in mice. Mice heterozygous for syntaxin 3 did not exhibit retinal degeneration in adulthood [44,82]. Furthermore, even mice as old as 26 months did not show evidence of increased photoreceptor loss when compared to age-matched controls [82]. Thus, although syntaxin 3 is required for the function and survival of photoreceptors, the loss of one syntaxin 3 allele does not increase the risk of age-related retinal degeneration.

That one functional syntaxin 3 allele is sufficient to maintain photoreceptor viability bodes well for gene therapy approaches. However, it should be kept in mind that while the focus in the mouse studies was on photoreceptors due to their dramatic degeneration, they are not the only retinal neurons that require syntaxin 3B for their function. Loss-of-function syntaxin 3 mutations that impact syntaxin 3B would also be expected to interfere with the ability of bipolar cells to release glutamate and thus, the transmission of visual information required for image formation to downstream retinal neurons and higher visual areas. Therefore, vision restoration strategies for patients with pathogenic syntaxin 3 mutations, or indeed any mutation that selectively impacts retinal ribbon-style synapse function, would necessitate the transduction of both classes of neuron with a functional or repaired gene.

## 6. Syntaxin 3B Functionality Is Regulated by Phosphorylation at T14

The domain structure of syntaxin 3B is similar to that of syntaxin 1 and is characterized by (from N to C terminus): the N-terminal domain comprising a short N-terminal sequence called the N-terminal peptide and three coiled coil domains that form the Habc domain, a flexible linker region, the SNARE (soluble N-ethylmaleimide-sensitive factor attachment protein receptor) domain, a short, polybasic juxtamembrane domain, and a C-terminal transmembrane domain (Figure 1C) [1,85,86]. As with syntaxin 1A, the N-terminal domain of syntaxin 3B is thought to fold over and occlude the SNARE domain, inhibiting syntaxin 3B from readily forming functional SNARE complexes [23,24]. For syntaxin 1, the transformation from the closed conformation to the open conformation is catalyzed by the arrival of Munc13 [86,87], and this is an essential step for neurotransmitter release at conventional synapses [87,88]. Surprisingly, inactivation of the gene that encodes for the only known Munc13 isoform expressed in adult photoreceptors does not hinder the ability of photoreceptors to release neurotransmitter [89]. This unexpected finding raises the possibility that retinal ribbon synapses possess an alternate pathway for syntaxin 3B activation.

**Figure 4:**
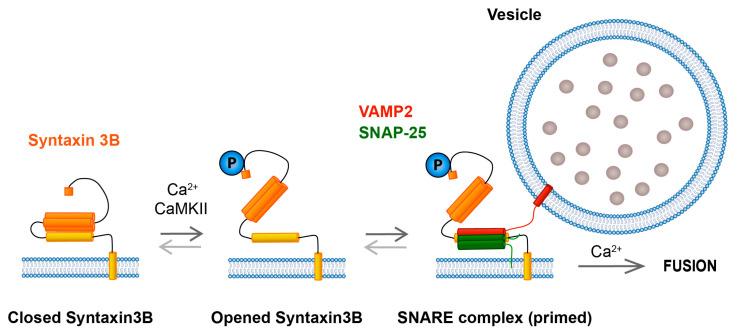
The calcium-dependent phosphorylation of syntaxin 3B at T14 by CaMKII facilitates SNARE complex formation. Syntaxin 3B preferentially exists in the closed conformation. Elevation of the intracellular presynaptic calcium concentration leads to the phosphorylation of syntaxin 3B at T14, located near the N-terminal peptide, via CaMKII. This phosphorylation step facilitates a conformational change in syntaxin 3B that exposes its SNARE-binding domain, enhancing the ability of syntaxin 3B to interact with SNAP-25 and VAMP2, priming the vesicle for rapid, calcium-triggered fusion. The three HA domains and the N-terminal peptide of syntaxin 3B are shown in orange, while the SNARE binding and transmembrane domains are shown in yellow. Phosphorylation at T14 is denoted by the blue circle containing the letter P. Note: Munc proteins have been omitted for better visualization of syntaxin 3B. For similar reasons, this model does not include other steps in neurotransmitter release that may be modulated by phosphorylation.

It has now become clear that the transition of syntaxin 3B from a self-inhibited, inactive closed conformation to an active, open conformation that can interact with SNARE binding partners is facilitated by the phosphorylation state of T14, located near the N-terminal peptide of syntaxin 3B (Figure 4). The importance of T14 phosphorylation for syntaxin 3B activation was first proposed ten years ago by Janz and colleagues [23]. This perceptive interpretation was based, in part, on the observations that syntaxin 3B lacking its N-terminal domain bound SNAP-25 more efficiently than syntaxin 3B with an intact N-terminal domain and that syntaxin 3B with a T14 phosphomimetic mutation bound SNAP-25 more efficiently than wild-type syntaxin 3B [23]. Consistent with a difference in conformation between phosphorylated and dephosphorylated syntaxin 3B at T14, the CD spectra of a T14 phosphomimetic mutant of syntaxin 3 and phospho-null syntaxin 3 were found to differ [90].

Perhaps the strongest confirmation that T14 phosphorylation facilitates the ability of syntaxin 3B to participate in SNARE-mediated membrane fusion comes from two recent studies. With the use of an elegant, single-molecule FRET-based approach, Gething and colleagues demonstrated that 90% of wild-type syntaxin 3B existed in the self-inhibited, inactive closed conformation [24]. By contrast, the T14E phosphomimetic mutant of syntaxin 3B readily sampled the open conformation such that the amount of protein in the closed conformation decreased by 50%, promoting the formation of syntaxin 3B-containing SNARE complexes with SNAP-25 and VAMP2 [24]. In the second study, performed using a highly controlled proteoliposomal fusion assay, the extent of membrane fusion between liposomes containing a target-SNARE (t-SNARE) complex comprising SNAP-25 and syntaxin 3B, with or without a T14 phoshomimetic mutation, and a liposome containing VAMP2 was quantified. Results revealed a stimulatory effect of the syntaxin 3B T14 phosphomimetic mutation on membrane fusion [14]. The effect was modest; however, the assay used may have underestimated the full impact of T14 phosphorylation on syntaxin 3B activation because the initial interaction between syntaxin 3B and SNAP-25 is thought to have occurred prior to the onset of measurement [14].

Phosphorylation of syntaxin 3B at T14 in the synaptic terminals of retinal photoreceptors and bipolar cells is light-regulated and calcium-dependent. With the use of an immunohistochemical approach and a phosphospecific T14 syntaxin 3B antibody, Campbell and colleagues demonstrated an increase in presynaptic T14 phosphorylation in mice under lighting conditions associated with membrane depolarization and increased neurotransmitter release, with photoreceptors having the highest level of phosphorylation in their terminals in dark-adapted mice and rod bipolar cells exhibiting the highest levels of phosphorylation in their terminals following the presentation of a light stimulus [29]. In ex vivo and in vitro studies, T14 phosphorylation driven by a light stimulus was shown to be dependent upon extracellular calcium, consistent with an activity-dependent process, while in isolated rod bipolar cells, elevation of intracellular, presynaptic calcium alone was sufficient to drive T14 phosphorylation [29]. The calcium-dependent phosphorylation of syntaxin 3 at T14 in the synaptic terminals of rod bipolar cells was reduced following application of a calcium/calmodulin-dependent protein kinase II (CaMKII) peptide inhibitor [29], a finding consistent with earlier in vitro biochemical studies that identified T14 of syntaxin 3 as a substrate of calcium/calmodulin-dependent protein kinase II (CaMKII) [23,91].

The T14 CaMKII regulatory site is unique to syntaxin 3 and is not present in syntaxin 1A [92]. Rather, residue S14 of syntaxin 1A is a substrate of casein kinase II and is believed to be constitutively phosphorylated [91,93,94,95]. In addition, experiments performed in hippocampal neurons have failed to show that S14 phosphomimetic and phospho-null mutants of syntaxin 1A impact neurotransmitter release [96]. By contrast, electrophysiological evidence obtained at bipolar cell and photoreceptor ribbon synapses support the enhancement of vesicle pool replenishment and exocytosis by calcium, calmodulin, and CaMKII [29,97,98,99], findings that are consistent with the syntaxin 3B T14 phosphorylation model. A cautionary note here is that pharmacological tools typically utilized can impact multiple downstream targets, and thus a more direct test of the effects of T14 phosphorylation is needed. Nevertheless, the evidence obtained thus far is consistent with the interpretation that the phosphorylation state of T14 and, hence, the ability to form syntaxin 3B-containing SNARE complexes is dynamically modulated in a light-regulated, calcium-dependent manner at retinal ribbon-style synapses.

What advantages might the activity dependence of syntaxin 3B activation provide for retinal signal processing? Given that syntaxin 3B is located throughout the plasma membrane of the synaptic boutons and is not restricted to the ribbon-style active zones [23] and that the synaptic boutons of photoreceptors and bipolar cells are filled with synaptic vesicles [38], a calcium-regulated activation step for SNARE complex formation could serve to restrict exocytosis to regions of elevated intracellular calcium (e.g., active zones) and reduce spontaneous fusion events and synaptic noise arising from more distal locations. Moreover, a calcium-regulated activation step would restrict intervesicular fusion and sequential quantal fusion to stimulation conditions that elevate intracellular calcium across multiple fusion sites or rows of a synaptic ribbon (e.g., Figure 3). In addition, this calcium-regulated activation step might contribute to, or even underlie, the calcium- and calmodulin-dependent acceleration of vesicle replenishment observed at these synapses [99,100] and support the ability of retinal ribbon-style synapses to continuously relay visual information across a wide range of visual stimuli. Finally, one cannot rule out the possibility that this mechanism could increase the number of SNARE complexes per vesicle, which in turn might alter fusion probability [66] and the precision of temporal coding.

## 7. Role of the Juxtamembrane Domain (JMD) of Syntaxin 3B in Exocytosis

The juxtamembrane domain (JMD) of syntaxin 3B has also been shown to have a role in the regulation of neurotransmitter release [101]. For syntaxin 1, the JMD has been suggested to facilitate the closed to open transition, the zippering of SNARE complexes, and the overcoming of the energy barrier for membrane fusion, at least in part, via a combination of electrostatic forces and palmitoylation [101,102,103,104,105,106]. A critical region within the syntaxin JMD is the short stretch of five polybasic amino acids. Syntaxin 3B and syntaxin 1A have a single amino acid difference in this key region. Recently, this naturally occurring difference has been shown to profoundly impact evoked and spontaneous neurotransmitter release in cultured hippocampal neurons [101]. Whereas the syntaxin 1 JMD contains the sequence “260-KARRKK-265”, the analogous syntaxin 3B sequence, “259-EARRKK-264”, was found to have a charge reversing substitution at the beginning of the sequence that replaces lysine (K) with glutamate (E). In syntaxin 1-null hippocampal neurons, the exogenous expression of wild-type syntaxin 3B was unable to rescue spontaneous and evoked neurotransmitter release. However, when the E at position 259 of syntaxin 3B was replaced with K to mimic the syntaxin 1A sequence, exogenous expression of the mutated syntaxin 3B construct rescued both spontaneous and evoked exocytosis [101].

The importance of residue 259 of syntaxin 3B at a retinal ribbon-style synapse has yet to be identified. However, evidence from syntaxin 1 suggests that the single amino acid difference in syntaxin 3B could impede palmitoylation of the syntaxin 3B transmembrane domain and lead to a reduction in spontaneous release [101] and improve the ability to detect light-driven events. Finally, it is interesting to note that the interaction of the JMD of syntaxin 1A with plasma membrane PIP2 has been suggested to regulate the accessibility of the N-terminus of syntaxin 1A to phosphorylation by casein kinase II at S14 [102,107,108]. Given that wild-type syntaxin 3B does not rescue transmitter release in hippocampal neurons but the E259K syntaxin 3B mutant does, one possibility is that this single amino acid regulates this plasma membrane interaction and, hence, the accessibility of the syntaxin 3B N-terminal T14 to phosphorylation by CaMKII. A task for the future is to test the above hypotheses regarding the role of the JMD and determine the extent to which the JMD impacts syntaxin 3B phosphorylation and function and the transmission of visual information at retinal ribbon-style synapses.

## 8. Regulation of Syntaxin 3B Function by Munc18

Munc18 proteins regulate SNARE-mediated membrane fusion [2,3,86,109]. At conventional synapses, Munc18-1 stabilizes the closed conformation until the arrival of Munc13 [2,3,109,110]. Once opened, syntaxin 1 binds to its SNARE binding partners. Munc18-1, along with Munc13, are then thought to help chaperone proper SNARE complex assembly [2,86,110]. In the retina, the importance of the syntaxin 3B–Munc18-1 interaction is highlighted by a recent study in human patients with congenital nystagmus linked to a mutation in Munc18-1 (pHis16Arg) [111]. This mutation enhanced the binding of Munc18-1 to syntaxin 3B [111]. By contrast, the binding to syntaxin 1A was not altered by the mutation [111], hinting at the possibility of a specific interaction interface between Munc18-1 and syntaxin 3B.

How might the pHis16Arg Munc18 mutation impact ribbon synapse function? We know that the binding of wild-type Munc18-1 to syntaxin 3B locks syntaxin 3B in the closed conformation [24]. Given the enhanced binding to syntaxin 3B by the mutant Munc18-1, mutant Munc18-1 may more strongly stabilize the closed configuration of syntaxin 3B relative to wild-type Munc18-1. This sequestration of syntaxin 3B due to enhanced binding with mutant Munc18-1 would be predicted to mimic a syntaxin 3B loss of function mutation with one crucial difference. While the latter fully blocks membrane fusion, the former would not. Two studies have shown that despite the stabilizing effect of wild-type Munc18-1 on the closed conformation of syntaxin 3B, syntaxin 3B phosphomimetic mutants are nevertheless able to overcome this inhibitory interaction to allow a syntaxin 3B T14 phosphomimetic mutant to sample the open state and bind SNARE partners [23,24]. In addition, SNARE-mediated fusion is enhanced to a greater extent in the presence of Munc18-1 when the SNARE complexes contain the T14 phosphomimetic syntaxin 3B mutant compared to wild-type syntaxin 3B [14]. Thus, although the reported human Munc18-1 mutation exhibits enhanced binding to syntaxin 3B, synapses may retain some level of functionality if syntaxin 3B is phosphorylated at T14.

## 9. Regulation of Syntaxin 3B Function by Complexin

Complexins 3 and 4 are members of a family of proteins that regulate SNARE-mediated exocytosis [86,112,113,114] that are preferentially expressed in the synaptic layers of the retina and, in particular, within the synaptic terminals of photoreceptors and bipolar cells [115,116]. Complexins 3 and 4 have been shown to have a clamping effect on membrane fusion mediated by syntaxin 3B-containing SNARE complexes [14] reminiscent of the clamping function reported for complexins 1 and 2 on membrane fusion mediated by syntaxin 1-containing SNARE complexes [117]. Manipulation of complexin 3 and/or 4 function in retinal ribbon-style synapses, with the use of a knockout approach or via the introduction of a peptide inhibitor of complexin function, has revealed that loss of complexin 3 and/or 4 activity results in enhanced spontaneous fusion and reduced evoked release [118,119,120], suggesting a role for complexins 3 and 4 in enhancing the signal-to-noise ratio of stimulus-evoked transmission of visual information. Interestingly, in cones, complexin 3 expression is regulated by the circadian clock [121], adding another dimension to the regulation of syntaxin 3B-mediated synaptic transmission. Floating or non-anchored structures were also reported in the absence of complexins 3 and/or 4 [122]. Similar defects have been reported in the absence of syntaxin 3B [44], SNAP-25 [79], and bassoon [37,123,124], highlighting the importance of the active zone macromolecular complex in the assembly, maintenance, and, ultimately, function of retinal ribbon-style synapses.

## 10. Concluding Remarks

That syntaxin 3 is required for vision is undisputed. Mouse models have shown that photoreceptors degenerate and die when the syntaxin 3 gene is inactivated, the major retinal splice form of which is syntaxin 3B, while human syntaxin 3 mutations that impact syntaxin 3B have been linked to an early-onset severe retinal dystrophy in patients with syntaxin 3A-associated microvillus inclusion disease. Mutations that selectively impair syntaxin 3B function are predicted to result in vision defects in the absence of gastrointestinal disease. Syntaxin 3B pairs with the conventional neuronal SNARE proteins SNAP-25 and VAMP2 to catalyze membrane fusion required for stimulus-evoked neurotransmitter exocytosis and the transmission of visual information from photoreceptors and bipolar cells to downstream neurons. Membrane fusion driven by syntaxin 3B-containing SNARE complexes is regulated by Munc18-1 and also by complexin proteins, albeit the latter are isoforms specific to retinal ribbon-style synapses and have their own interesting features. A particularly enigmatic aspect of syntaxin 3B is that its ability to form SNARE complexes is modulated by the phosphorylation state of T14, which in turn is regulated by light via a calcium/CaMKII-dependent pathway. Why retinal ribbon-style synapses have evolved this activity-dependent mechanism to regulate SNARE complex formation is not yet clear, although several hypotheses have been put forth. Whether this mechanism abrogates the need for Munc13, an essential priming factor of conventional synapses, has yet to be demonstrated. An important task for the future will be to test these hypotheses and establish the impact of syntaxin 3B and its post-translational modification(s) on protein–protein interactions, synaptic vesicle dynamics, neurotransmitter release, and the transmission of visual information across the vertebrate retina. An additional charge for the future will be to develop methods to exploit the unusual calcium-dependent regulation of syntaxin 3 functionality for therapeutic gain in retinal disease and in disorders of other syntaxin 3-expressing cells.
